# High performance adaptive maximum power point tracking technique for off-grid photovoltaic systems

**DOI:** 10.1038/s41598-021-99949-8

**Published:** 2021-10-14

**Authors:** Fahd A. Banakhr, Mohamed I. Mosaad

**Affiliations:** Electrical and Electronics Engineering Technology Department, Yanbu Industrial College (YIC), Yanbu, Saudi Arabia

**Keywords:** Electrical and electronic engineering, Energy science and technology, Engineering

## Abstract

Solar photovoltaic (PV) energy has met great attention in the electrical power generation field for its many advantages in both on and off-grid applications. The requirement for higher proficiency from the PV system to reap the energy requires maximum power point tracking techniques (MPPT). This paper presents an adaptive MPPT of a stand-alone PV system using an updated PI controller optimized by harmony search (HS). A lookup table is formed for the temperature and irradiance with the corresponding voltage at MPP (V_MPP_). This voltage is considered as the updated reference voltage required for MPP at each temperature and irradiance. The difference between this updated reference voltage at MPP and the variable PV voltage due to changing the environmental conditions is used to stimulate PI controller optimized by HS to update the duty cycle (D) of the DC–DC converter. The temperature, irradiance, and corresponding duty cycle at MPP are utilized to convert this MPP technique into an adaptive one without the PI controllers' need. An experimental implementation of the proposed adaptive MPPT is introduced to test the simulation results' validity at different irradiance and temperature levels.

## Introduction

Due to the environmental difficulties and the significant increase in energy demands, there has become a global and urgent need to generate power from renewable energy sources (RESs). This urgent need to use and implement RESs is due to several factors, including but not limited to clean and non-polluted production of energy, continuity generations as these RESs will not be depleted. Many RESs with significant penetration into the electrical networks were used and implemented based on many RESs types. Wind energy, fuel cell and photovoltaic (PV) were used in many applications, either on or off-grid applications^1–3^. Some of these RES may be connected to form hybrid RES. These hybrid systems are used for on and off-grid applications^4^.

PV systems are widely spread as one of the RESs. Growth of PV systems and solar energy PV systems had become common in many off and on-grid applications with expected generating level PV up to 1 TW in 2021^5^.

The initial cost and the subsequent generating unit (kW.hr) cost of PV systems is still relatively high compared to the classical fuel. This increase in costs has led to a significant tendency to use the available output power from PV without directions to create new PV systems, if possible. One of the well-known techniques for using the available power extracted from PV systems is maximum power point tracking (MPPT).

MPPT of PV systems means controlling and adapting the output of generated power from PV to MPP available from PV at certain surrounding environmental conditions. MPPT is based on control and drive the duty cycle (D) of the DC-DC converter connecting the DC output of PV system, feeding the load in off-grid applications or feeding an inverter in on-grid applications. A new PV-MPPT based on the control of the DC-DC chopper as in Ref.^6^. A modified adaptive hill climbing PV-MPPT was introduced in Ref.^7^. A sensorless single-cell MPP for vehicle solar arrays applications was proposed^8^. The parallel connection of PV systems for standalone configuration was modeled and conducted in Ref.^9^. A comparison between different MPPT techniques was presented in Ref.^10^.

The output power of the PV system mainly relies on the surrounding temperature and the irradiance. MPPT techniques are to force the PV system's characteristics to operate at the MPP to exploit the available energy from PV at a certain temperature and irradiance conditions by regulating the DC–DC converter's duty cycle. Fractional open circuit and fractional short circuit, the conventional solutions for MPPT were presented, but they were not accurate to track the maximum operating point^11^. Different MPPT algorithms were proposed; from them, perturb and observation (P&O) was used in many applications^3^. P&O is a very simple technique and easy to be implemented^12^. P&O is affected mainly by the perturb step value for both transient and steady-state operating conditions. That calls for adaptive regulation of the perturbation step as in Refs.^13,14^. Different procedures were applied to convert the MPPT into adaptive MPPT^15–18^. These adaptive techniques suffered from high computational load and the users' well-known to adapt the perturb step size.

Incremental conductance (IC) method with endeavors to overcome the problems that appear when using P&O MPPT technique was presented in Refs.^19–21^. IC gave a better performance than P&O in the transient periods associated with rapid changes in the environmental conditions. Some modifications are applied to improve the IC method by adjusting PI controllers with the capability to modify the duty cycle of the DC–DC converters. The challenges in these modifications are to determine the PI controller parameters and how to adapt them quickly with variations in the environmental conditions. Many other artificial techniques were presented to overcome the MPPT problems that emerged when using P&O and IC techniques. Fuzzy logic controller is proposed to update the control signal instead of a fixed signal when using static PI controller parameters for PI controllers^22^. Another artificial technique is used to update the duty cycle control signal to achieve the MPPT of PV system, artificial neural network^23,24^. Despite the advantages of using both Fuzzy logic controllers and artificial neural network^22–24^, over static PI controllers, they are more complex than PI controllers, along with their implementation difficulty. Besides, fuzzy logic controller was used alone and a hybrid with firefly for achieving MPPT^25–27^.

For tuning the PI controller parameters, many optimization techniques were introduced. Genetic algorithms, particle swarm optimization, cuckoo search and harmony search (HS), elephant herding optimization were introduced to optimize PI controller parameters for many power systems applications^28–32^. Hybridization of two or more optimization techniques was presented for MPPT. Hybrid PSO and gray-wolf optimization techniques was presented and compared to single optimization techniques for MPPT^33^. Another hybrid optimization technique based Jaya optimization is proposed for MPPT in Ref.^34^. These optimization techniques succeed at the optimal tuning of the PI controller parameters off-line. With varying operating conditions, the optimized PI controller parameters could not condemn the optimality, if they are not returned. Adaptive algorithms should be introduced to convert PI controller into an optimal one. Artificial neural network was introduced to convert adaptive PI controllers into adaptive one as in Ref.^34^. Another adaptive control technique was presented to convert the PI controller parameters (static gains) into adaptive ones through adaptive reference PI controllers. This adaptive control was presented to enhance the PV system performance by updating the voltage reference based on the variable environmental operating conditions^35^. However, these techniques acquire high experience for users, and much training data to assure the systems' accuracy.

In this paper, a robust and straightforward adaptive MPPT technique is proposed. The PV curve of the PV system is plotted and verified practically. A lookup table is formed for different values of irradiance, temperature and the corresponding voltage at MPP (VMPP_ref). This MPP voltage is taken as the updated reference and compared to the varied PV voltage due to the variation of irradiance and temperature. The error between the reference updated voltage and the PV voltage represents the input to the PI controller. This error is minimized using HS optimization. This process is repeated 30 times to obtain the optimized PI controller parameters with the corresponding duty cycles required for MPPT at each temperature and irradiance. The irradiances, temperatures and the corresponding optimized duty cycles required for MPPT are used achieve MPPT at each operating condition. With these data (irradiance, temperature and the duty cycle), the system is converted to adaptive MPPT without using PI controller. These data were used to build up a practical circuit to achieve the adaptive MPPT technique proposed. A simple microcontroller is used with the second table data to dive the DC-DC converter with the updated duty cycle at each irradiance and temperature to assure adaptive MPPT at different temperatures and irradiances.

## Problem formulation

**Figure 1:**
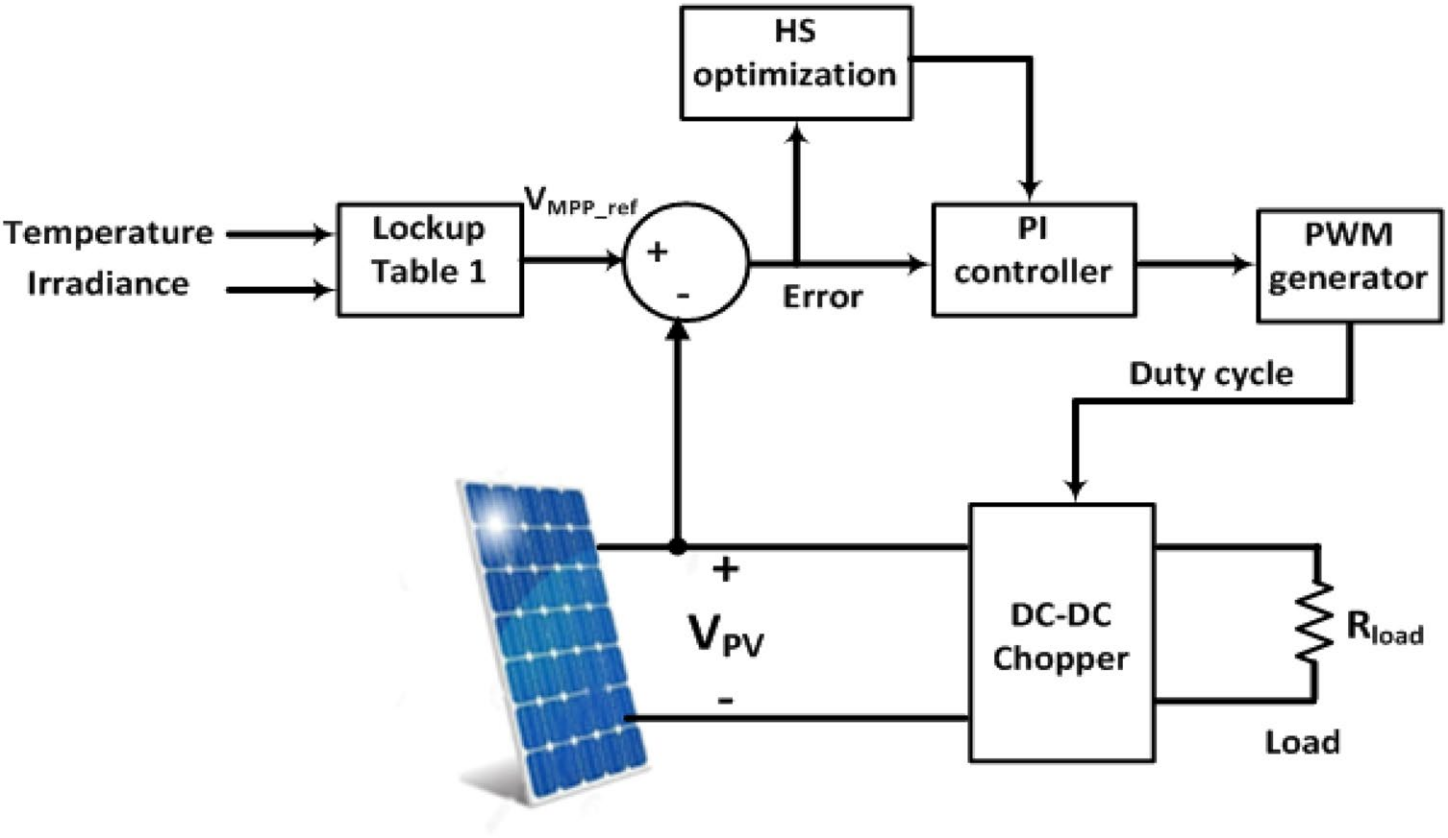
System under study.

The proposed system, Fig. 1, comprises a PV module connected to a DC–DC converter feeding a load. The DC–DC converter's duty cycle ratio is controlled using an optimized PI controller to maintain MPP of the PV system. A lookup table (Table 1) consists of the temperature, irradiance and the corresponding voltage at MPP obtained from the characteristics is formed. This table is used for updating the reference voltage (VMPP_ref) for MPP at each temperature and irradiance. The PV voltage (Vpv) is sensed and compared to the updated reference voltage. This error is minimized by HS optimization technique and used to stimulate the DC-DC chopper to track MPP^36–39^.

**Table 1:** Specifications of EBS solar module at standard conditions.

Open circuit voltage	V_OC_	21.8 V
Short circuit current	I_sc_	2.23 A
Voltage at max. power	V_m_	17.2 V
Current at max. power	I_m_	2.04 A
Maximum power	P_m_	35 W

### PV modeling

**Figure 2:**
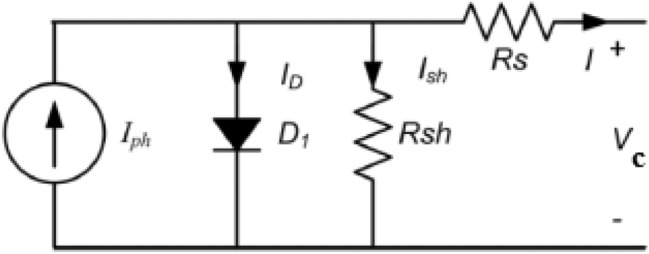
Solar cell equivalent circuit.

The PV equivalent circuit is given in Fig. 2. This circuit comprises a light-dependent current source with a shunted diode^6,7^. The current produced by this current source is directly proportional to the light dropped on the PV. R_S_ and R_p_ are series and shunt resistances of PV, respectively.

In this paper, the EBS solar module is used and the specifications of this module are given in Table 1 at standard conditions of 25 °C temperature and irradiance of 1000 W/m^2^.

### PI controller design

The updated reference voltage, V_MPP_ref_ represents the voltage at which MPP is attained is used and compared to the current PV voltage, V_pv_. The difference between these two voltages ev represents the error used to drive PI controller. An objective function, J is formed based on the integral square of error of ev that can be defined as:

1 $$ {\text{J}} = \int\limits_{0}^{t} {(({\text{ev}}({\text{t}}))^{2} } \;{\text{dt}} $$

The HS optimization technique is used to minimize this objective function and determine the optimal PI control parameters at each irradiance and temperature.

### Harmony search optimization

The first step in HS is the initialization of the two PI control parameters by supposing random values of them. Then the fitness function J, given in (1) is determined with these random values. Then the Harmony Memory (HM) is set as in (2)

2 $$ HM = \left[ {\begin{array}{*{20}l}    {K_{p}^{1} } \hfill & {K_{i}^{1} } \hfill  \\    {K_{p}^{2} } \hfill & {K_{i}^{2} } \hfill  \\     \vdots  \hfill &  \vdots  \hfill  \\    {K_{p}^{{HMS}} } \hfill & {K_{i}^{{HMS}} } \hfill  \\   \end{array} } \right] $$

The HM matrix contains two columns for the PI controller parameters (K_p_, K_i_). Each row in this matrix is the enhanced harmony vector based on the harmony memory considering rate (HMCR) and pitch adjusting rate (PAR). The updated harmony vector ${x}_{i}^{n+1}$ of the current harmony vector ${x}_{i}^{n}$ is calculated as:

3 $${x}_{i}^{n+1}={x}_{i}^{n}+rand*BW$$

where *rand* is a random value between (0, 1) and *BW* is an arbitrary distance bandwidth.

**Figure 3:**
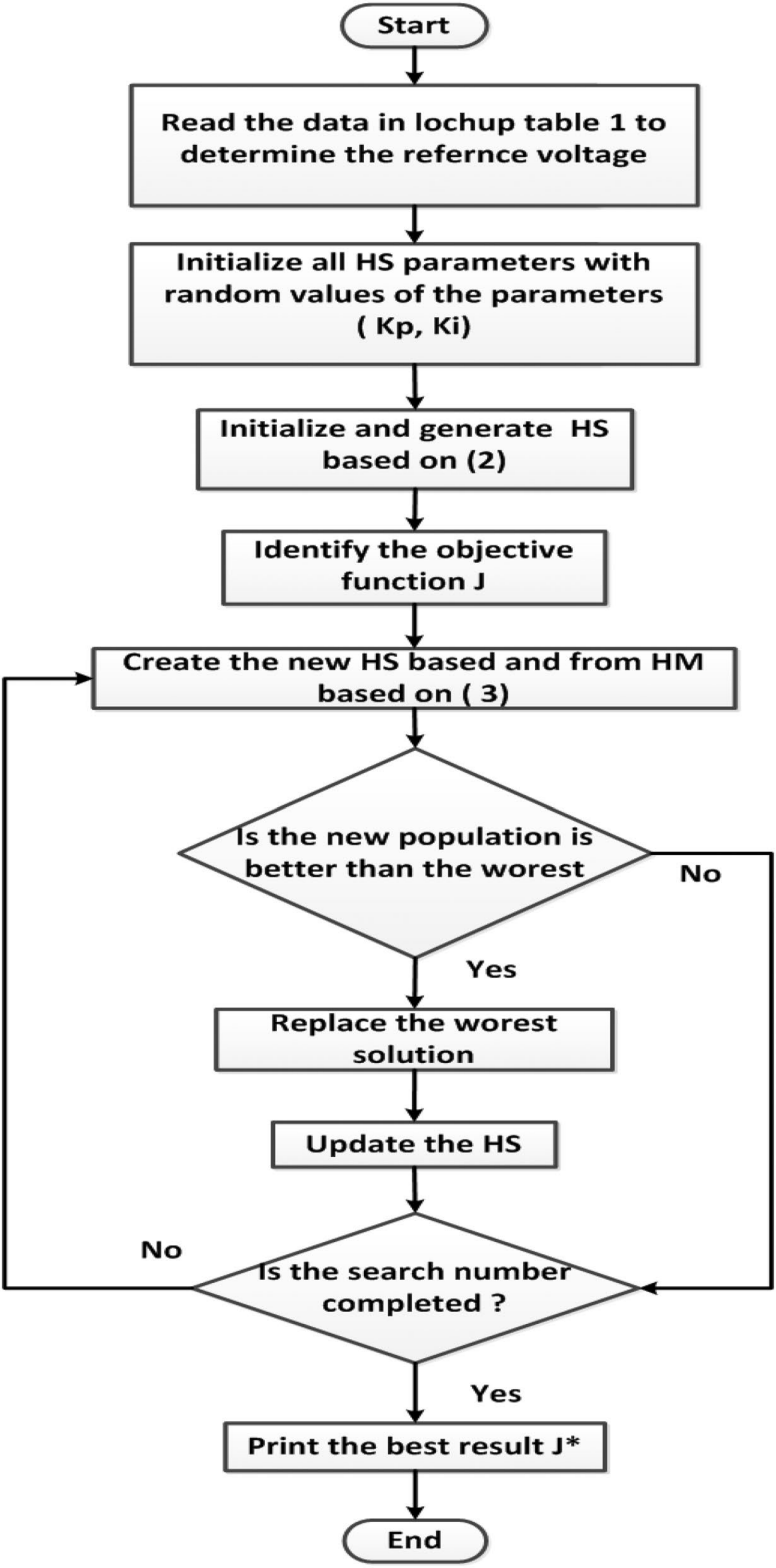
HS optimization flow chart.

The updating process is done again till reaching the minimum possible value for the objective function *J* or reaching the maximum search iteration numbers, Fig. 3^36^.

## Simulation results

### Harmony search optimization technique

**Figure 4:**
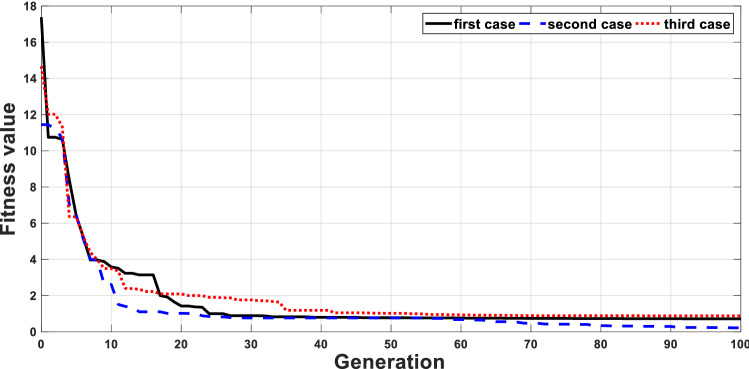
Convergence of objective function using HS.

Due to the temperature and irradiance variations, the PV output voltage will change to new values different from the required values for MPP. These variables PV voltages will be compared to the voltage required for MPPT at these environmental conditions as in Table 2. The error between the PV voltage and the updated reference voltage based on Table 2 is used to drive PI controller to produce the duty cycle required for the converter. The HS optimization algorithm is used to minimize the objective function given in (1) and then determine the optimal PI controller parameters. HS optimization is used 30 times to obtain the optimal PI controller parameters for each case. The convergences of the first three cases are given in Fig. 4.

**Table 2:** Temperature, irradiance PV voltage and power at MPP and the control parameters.

Point	Tem (°C)	Irra (W/m^2^)	V_MPP_ (V)	P_MPP_ (W)	Optimized PI controller	
					K_p_	K_i_
1	22	200	16.470	5.5959	13.6819	40.191
2	25	200	16.261	5.5367	8.0478	39.300
3	28	200	16.0533	5.4764	9.6567	15.231
4	31	200	15.7406	5.4153	8.0437	10.100
5	34	200	15.5321	5.3533	12.4134	32.083
6	37	200	15.3236	5.2901	3.0874	18.891
7	22	400	17.3042	13.159	7.6269	18.107
8	25	400	16.9915	12.9979	3.2227	16.234
9	28	400	16.783	12.8354	15.1622	44.448
10	31	400	16.5745	12.6713	17.4222	28.625
11	34	400	16.2618	12.5065	7.0155	19.456
12	37	400	16.0533	12.3411	13.7107	18.919
13	22	600	17.5127	20.7386	5.8830	14.178
14	25	600	17.2	20.4769	10.6126	34.590
15	28	600	16.9915	20.2138	16.6485	37.350
16	31	600	16.783	19.9486	11.9498	26.869
17	34	600	16.4703	19.6823	6.7062	27.971
18	37	600	16.2618	19.4163	5.9845	37.564
19	22	800	17.5127	28.1916	9.0519	21.695
20	25	800	17.3042	27.8289	8.452	29.513
21	28	800	16.9915	27.467	7.19	16.450
22	31	800	16.783	27.1036	11.16	40.045
23	34	800	16.5745	26.7383	14.85	22.717
24	37	800	16.366	26.371	8.486	12.548
25	22	1000	17.408	34.923	8.587	19.535
26	25	1000	17.2	35	2.497	13.986
27	28	1000	16.991	34.539	9.488	31.442
28	31	1000	16.783	34.076	5.803	15.047
29	34	1000	16.4703	33.6139	6.3504	22.051
30	37	1000	16.2618	33.1505	13.0738	29.685

The optimized PI controller parameters using HS for the 30 cases proposed are given in Table 2.

### Adaptive maximum power technique proposed

**Figure 5:**
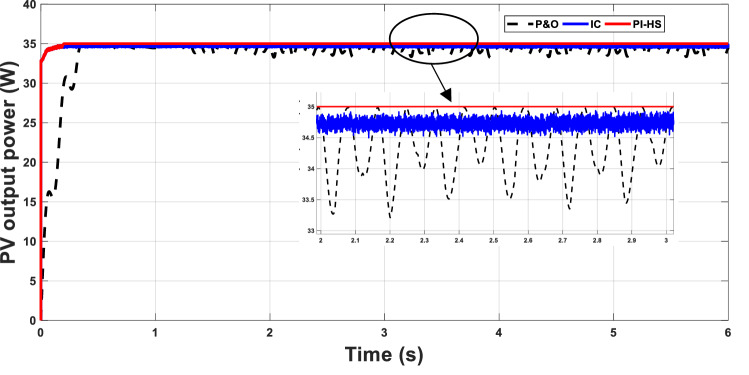
MPP at 25 °C and 1000 W/m^2^.

**Figure 6:**
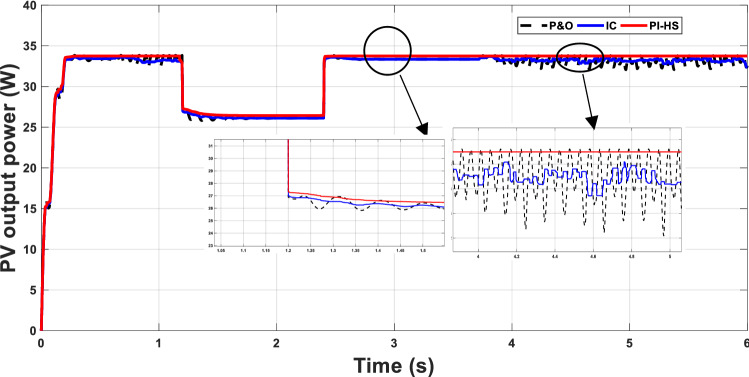
MPP with change from point 30 to point 22, defined in Table 2.

The proposed technique PI-HS for MPPT will be compared to P&O and IC methods to study the proposed method's effectiveness. At the standard conditions of 25 °C and 1000 W/m^2^ (point 26 in the table), the MPP with P&O, IC and PI-HS are plotted in Fig. 6. The performance of the PV system with the proposed technique surplus the performance with P&O and IC methods. There are fluctuations between 33.4 and 34.9 W in power generated from the PV were obtained when using P&O; slight improvement in these fluctuations was attained when using IC as in Fig. 5. The proposed HS-PI reached to the maximum power value of 35 W without any fluctuations.

The performance of the proposed system will be investigated with some changes in environmental conditions. A sudden change in the temperature from 37 to 31 °C and a sudden change in irradiance from 1000 to 800 W/m^2^ (change from point 30 to point 22 in Table 2) occurs at 1.17 s. The proposed technique gives better performance than P&O and IC techniques in both steady-state and transient intervals, Fig. 6.

The PI controller introduced in this article succeeded in achieving the MPPT with better performance. Another test case will be presented to check if the optimal PI controller parameters determine at certain conditions will satisfy the optimality at other operating conditions or not.

**Figure 7:**
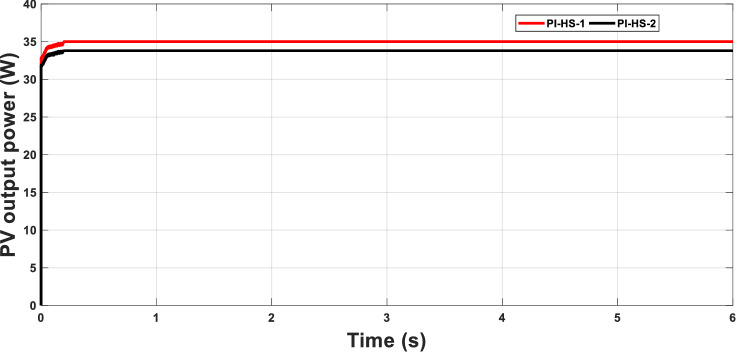
MPPT with fixed and variable PI-HS.

The standard test case (point 26) will be tested with two different values for the optimal PI controllers. The first PI controller, PI-HS-1 is the optimal controller parameters determined at the standard condition (2.497, 13.986), while the second controller PI-HS-2 is the PI control parameters at another point, point 24 as an example, with PI controller parameters of (8.486, 12.548). The PI controller parameters obtained for this case showed better performance and tracking the MPP to a higher level when using PI-HS-1 at which the operating conditions are considered than using PI-HS-2. This calls for changing the PI control parameters for each operating condition Fig. 7. In other words, convert the fixed PI controller parameters into variable ones based on the environmental conditions. This transformation is difficult to be applied practically. This issue had been addressed by adding another technique to the system and implement it practically.

## Testing the proposed MPPT algorithm

### Experimental verification of P–V and I–V characteristics

**Figure 8:**
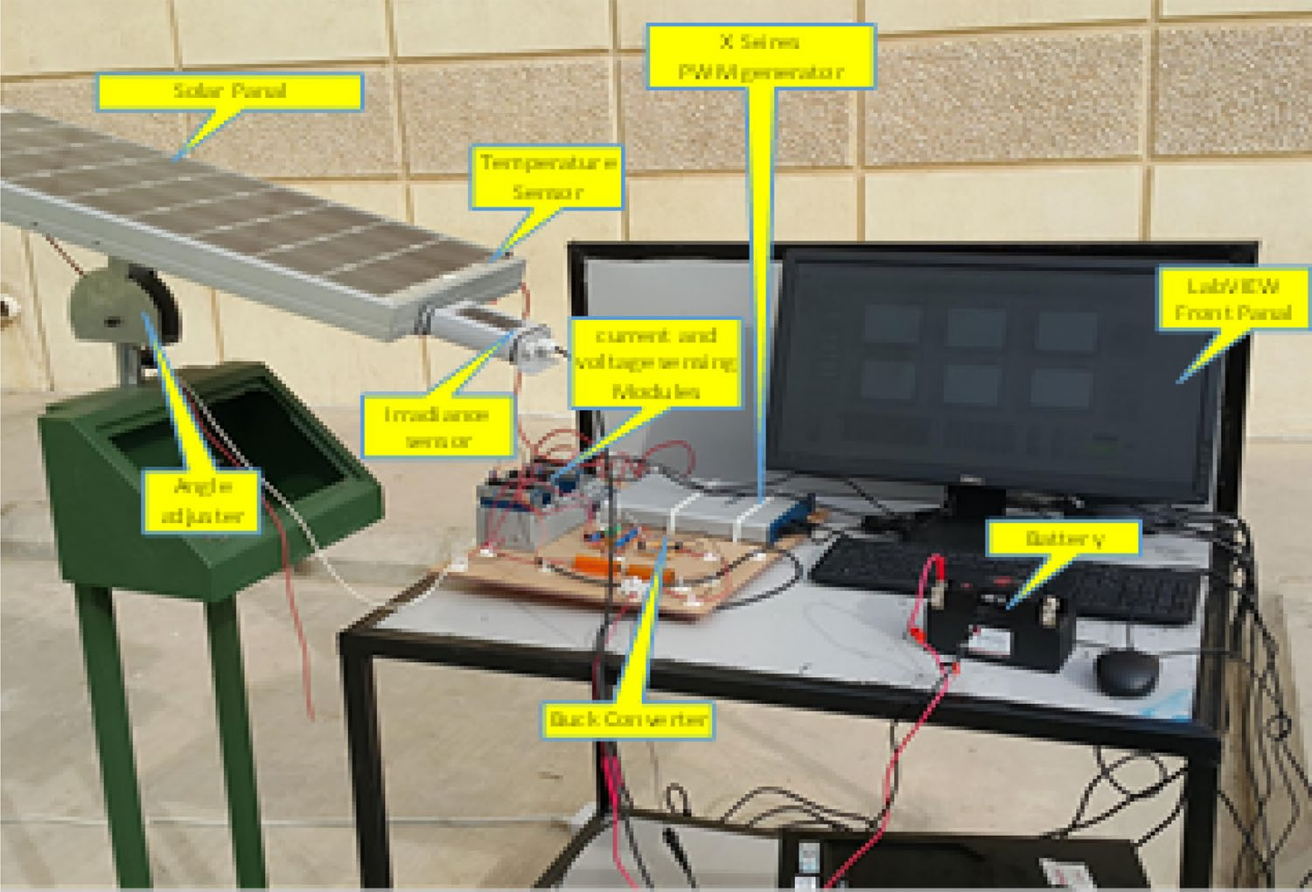
Experimental test ring.

Experimental test rig outdoor atrium in is shown Fig. 8. A solar panel is mounted at an optimum angle to get the maximum light at peak hours. An irradiance sensor is mounted at the same angle as the solar panel to get an accurate response. A temperature sensor is placed on the solar panel to get the panel's current temperature and find out its dependency on the temperature.

**Figure 9:**
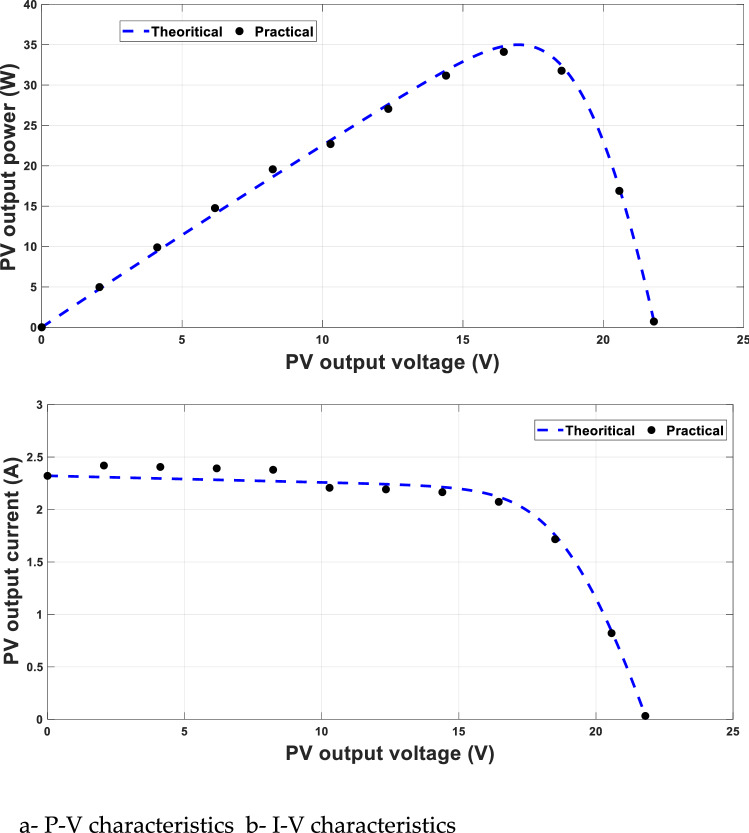
Experimental verification of solar cell characteristics.

The experimental verification of P–V and I–V characteristics of the solar cell used is given in Fig. 9a,b at 25 °C and 1000 W/m^2^. This experimental verification is used to verify the simulation and will be used many times at different environmental conditions to determine the voltage at MPP, which is, in turn, the updating the reference voltage (V_MPP_ref_).

The experimental set-up contains a solar panel, sensors for temperature and irradiance, buck converter, PWM generation and lab-view front panel. The updated duty cycle at MPP and the corresponding temperatures and irradiances are used and added to the Lab-view. At any values of irradiances and temperatures, the optimized duty cycle at MPP is the output from the PWM generation to make the PV system operates at the MPP conditions.

### Adaptive MPPT verification

The model is simulated for 30 cases, and each case has a certain temperature and irradiance to get the voltage at MPP from the P–V characteristics. The temperature is taken from 22 to 37 °C with a step of 3 °C while the irradiance is taken from 200 to 1000 W/m^2^ with a step of 200 W/m^2^, Table 2. From these data, the reference voltage is updated at each irradiance and temperature.

The modification technique is applied practically by determining the duty cycle required for MPPT corresponding to each operating condition. In other words, the output of PI-HS is determined at each point in Table 2. A new table is formed with the temperature, irradiance and the corresponding duty cycle. This technique is working without using PI controllers, which simplify the system and make it easy to implement.

**Figure 10:**
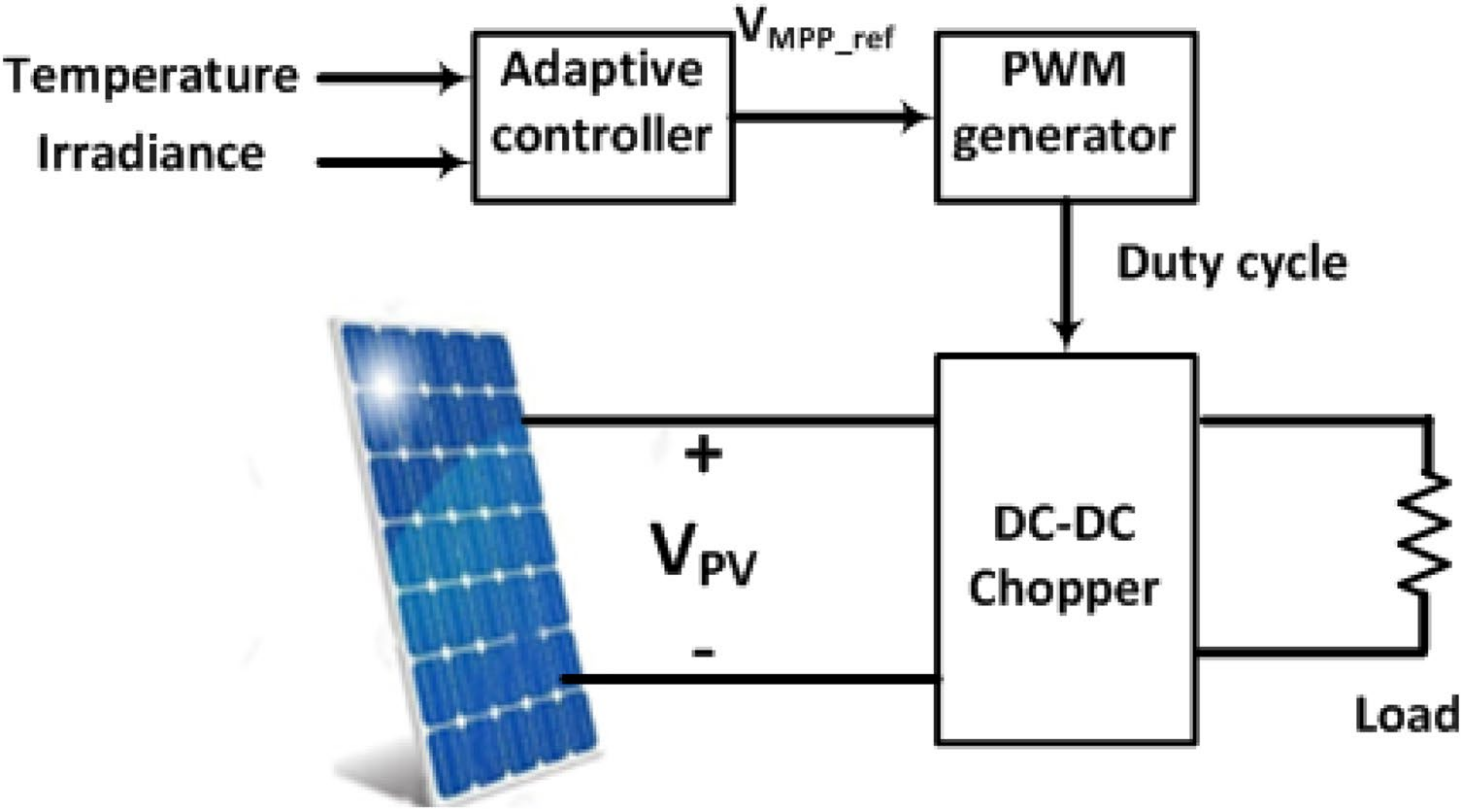
Block diagram of setup system.

In order to test the proposed technique for adaptive MPPT, a test setup was developed that was capable of data acquisition in real-time with parameter recording functionality. The block diagram of the system is shown the Fig. 10.

The block diagram shows the logical connections of different blocks of the system. Si-420TC irradiance sensor is used to sense light, which provides 4–20 mA current output, and is compensated by a built-in thermal compensator. Moreover, it has a wide range of spectral responses from 0 to 1200 W/m^2^. Besides, three-wire RTD is used to monitor solar panels' temperature in a wide range with high accuracy. Furthermore, National Instruments robust and precise current and voltage sensing modules NI9217 and NI9225 are used to monitor input and output parameters. PWM National Instrument X-series card is used for high frequency, which has 100 MHz on board time base for accurate duty cycle control and frequency generation.

**Figure 11:**
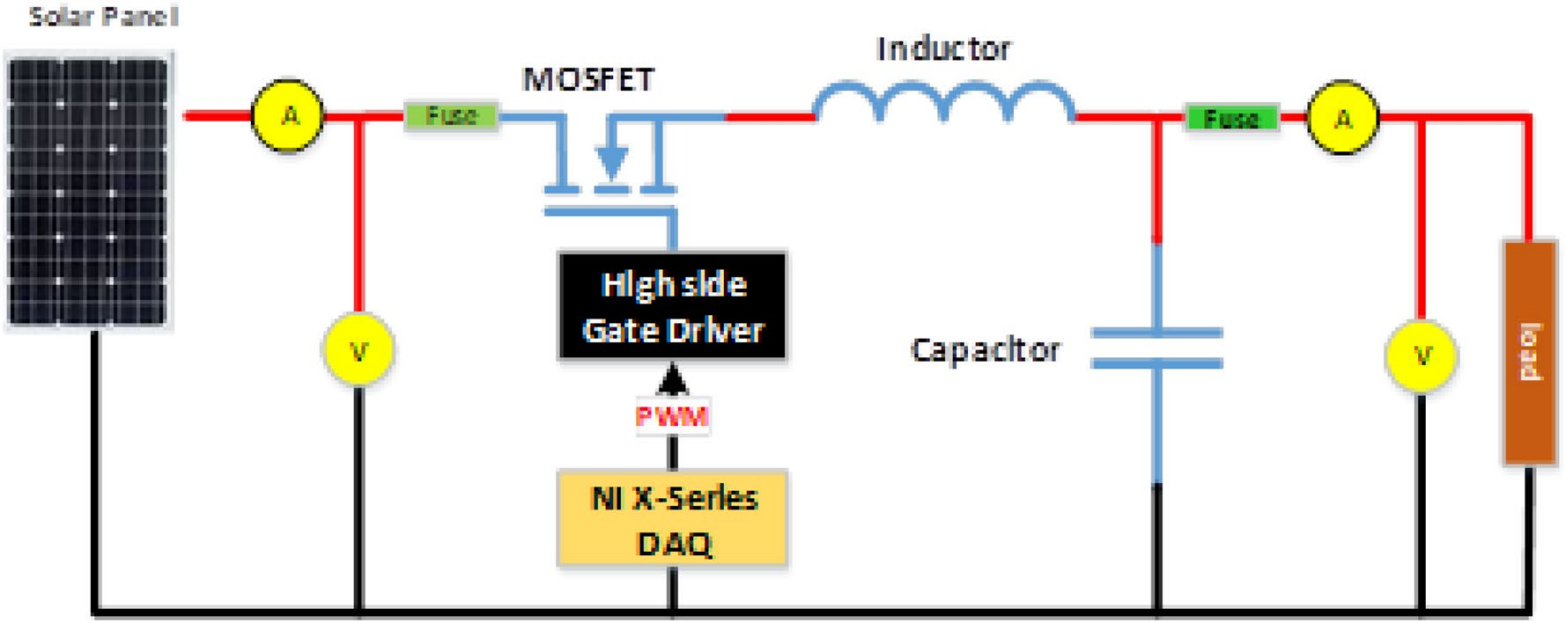
MOSFET switching circuit.

The switching circuit is based on a single MOSFET DC-DC buck topology. N-Channel MOSFET is used on high side. Since the gate is at a higher voltage level, a gate driver circuit is used to drive the MOSFET with respect to floating ground. Line fuses are used on both sides of the circuit for protection. A voltage sensor was placed to put recording the input and output values. The schematic is shown in Fig. 11.

The list of components used along with their values is given in Table 3.

**Table 3:** List of components for buck convertor.

Parts	Manufacturer	Value/part number
Inductor	Murata	1 mH
Capacitor	Murata	1 uF
MOSFET	International rectifier	IRF3205
Load	Bourns	Variable
MOSFET driver	International rectifier	IR2102

LabVIEW is used to develop the main software to test the proposed adaptive algorithm. LabVIEW Front panel shows input and output voltage; temperature, irradiance and power.

**Figure 12:**
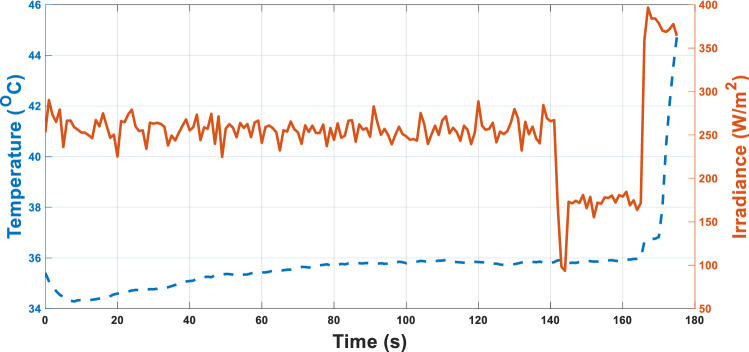
Temperature and irradiance variation in 1-day morning.

The test ring is left for 1 day for about 3 min outdoor, the irradiance and the temperature are recorded as in Fig. 12 using the temperature and irradiances sensors used in the experimental setup shown in Fig. 8.

The system is tested with the proposed adaptive MPPT with an adaptive duty cycle and compared to a fixed duty cycle for driving the DC–DC chopper.

**Figure 13:**
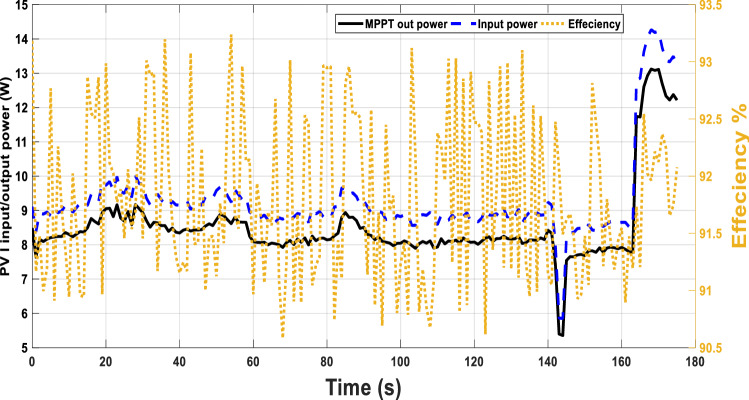
Adaptive MPPT with temperature and irradiance variation in one-day morning with efficiency.

The input and output power for the same environmental conditions, Fig. 13 are given in Fig. 13. The system efficiency with this adaptive duty cycle is improved with values greater than 90%.

**Figure 14:**
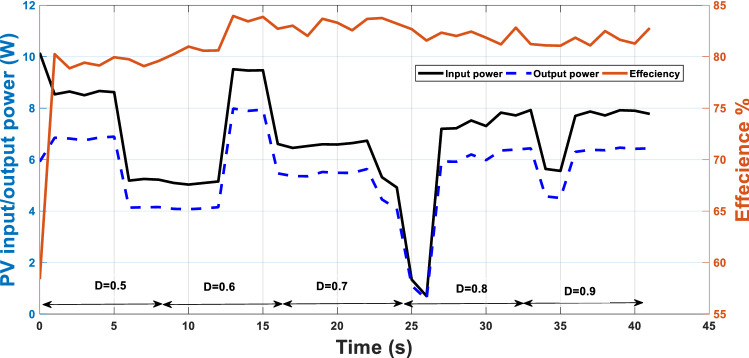
Fixed MPPT with temperature and irradiance variation in one-day morning with efficiency.

Another practical test case for fixed duty cycles is applied and the efficiency of the system will be investigated. For a certain period, the input, output power of PV and the efficiency are recorded. The duty cycle in this case is kept at fixed values not adapted according to the proposed technique. Five values of duty cycles of 0.5, 0.6, 0.7 0.8 and 0.9 are adjusted at each interval, Fig. 14. The system’s maximum efficiency is 84% compared to the range of efficiencies between 91 and 93% when using the adaptive duty cycle as in Fig. 13.

## Conclusions

This paper introduced a new adaptive MPP technique for a standalone PV system. This technique is based on updating the reference voltage for MPPT based on the environmental changes including irradiance and temperature. This updated voltage is compared to the PV output voltage and the error is used to drive the DC–DC converter. This error is minimized by harmony search optimization technique. The proposed technique updated the PI controller parameters and consequently the duty cycle required for the converter. The proposed adaptive technique gave a better performance than P&O and IC techniques in terms of system efficiency. An experimental setup is used to simplify the controller by lookup table consisting of the temperature, irradiance, and corresponding duty cycle required for each operating condition to achieve MPPT. Compassion between the proposed adaptive and some fixed values for the duty cycles necessary for MPPT was introduced at every operating point. This comparison proved the effectiveness of the proposed method in MPPT with higher efficiencies.

## Abbreviations

*BW*: Arbitrary distance bandwidth
D: Duty cycle
ev: Error in the voltage between PV voltage and the reference value
HM: Harmony memory
HMCR: Harmony memory considering rate
IC: Incremental conductance
I_sc_: Short circuit current
I_m_: Current at max. power
K_p_, K_i_: PI controller parameters gainn
MPPT: Maximum power point tracking
P_m_: Maximum power
PV: Photovoltaic
P&O: Perturb and observation
PI: Proportional-integral contoller
PAR: Pitch adjusting rate
RES: Renewable energy sources
*rand*: Random value between (0, 1)
RS: Series resistances of PV
Rp: Shunt resistances of PV
V_OC_: Open circuit voltage
V_m_: Voltage at max. power
VMPP_ref: Updating the reference voltage
